# Estimation of vehicle-induced bridge dynamic responses using fiber Bragg grating strain gages

**DOI:** 10.1177/0036850419874201

**Published:** 2019-09-29

**Authors:** Feng Xiao, Dejian Meng, Yang Yu, Yong Ding, Lijun Zhang, Gang S Chen, Wael Zatar, J Leroy Hulsey

**Affiliations:** 1 Department of Civil Engineering, Nanjing University of Science and Technology, Nanjing, China; 2 School of Automotive Studies, Tongji University, Shanghai, China; 3 College of Information Technology and Engineering, Marshall University, Huntington, WV, USA; 4 Department of Civil and Environmental Engineering, University of Alaska Fairbanks, Fairbanks, AK, USA

**Keywords:** Fiber Bragg grating strain gage, vibration monitoring, bridge health monitoring, dynamic feature identification, vehicle load

## Abstract

Strain gage sensors have been used to evaluate the local behavior of structures; however, there are limited studies for its application in bridge dynamic feature identification. In this study, fiber Bragg grating strain gages were installed on the lower chord members of a bridge, and dynamic features were identified successfully using strain gage readings when vehicles passed over the bridge. The results were also verified using a finite element model. The innovation presented in this article is the use of fiber Bragg grating strain gage readings to identify the dynamic features of a long-span, steel-girder bridge. To clarify the effect of truck dynamic load, the load spectrum of the truck is characterized. This article introduces a new method for identifying the dynamic parameters of bridges.

## Introduction

In bridge health monitoring, monitoring of the dynamic response of the bridge can provide important data, such as the natural frequency, damping, or mode shape, which can be used for bridge model updating or damage identification.^1–8^ Traditionally, to monitor the dynamic responses of a bridge, a fiber Bragg grating (FBG) accelerometer,^9^ a force balanced accelerometer,^10,11^ or a wireless accelerometer^12,13^ are employed. However, these types of sensors can only provide acceleration, and additional sensors are required to monitor local information regarding the bridge, such as strain and displacement. With advances in sensing technology and enhanced sensitivity, strain gages, displacement sensors, and tiltmeters have been used for dynamic identification.^14–17^ The sensors can not only provide the strain, displacement, or tilt but also have the capability to work as an accelerometer to monitor dynamic responses and to be used for dynamic feature identification.

The dynamic features of a structure can be estimated using strain gage signals. In comparison to using traditional accelerometers, using FBG strain gages advances the approaches used for structural dynamic feature identification. First, it is suitable for the structural health monitoring system that required many stain gages to estimate the structural deformation. Due to the multiplexing capability of the strain gage, the strain sensor can also record dynamic movements, which can be used to quantify dynamic features. The measured strain enables both local and global analyses of the bridge structure. Second, the FBG sensor has several advantages over traditional sensors,^18,19^ including stability, a nonconductive nature, and convenience.

Dynamic parameters have been successfully identified in laboratory tests for a steel beam, a reinforced concrete beam,^20,21^ an aluminum beam,^22^ an experimental pipe,^23^ and a bridge model.^24–26^ However, monitoring of the dynamic response of a long-span bridge using an FBG strain gage is lacking. In addition to monitoring local behavior, this study uses strain gages to monitor dynamic bridge responses at a global level. The innovation of this study is the use of FBG strain gage readings to identify the dynamic features of a long-span, steel-girder bridge.

## FBG strain gage technology

In this study, three FBG strain gages were used to identify the dynamic response of a bridge. FBG sensors are electromagnetic interference-resistant, corrosion-resistant, lightweight, small, and easily installed, and have been widely applied in bridge health monitoring.^27^ The structural health monitoring system^28^ used the os3155 strain gage.^29^ The os3155 includes a second FBG that provides active temperature compensation. The benefits of this approach include more accurate temperature compensation and a lower installation cost. The sensitivity of the strain gage is approximately 1.2 pm/µε, the operating range is −40°C to 80°C, and the strain limit is ±2500 µε.^29^ The sampling rate of the FBG strain gage was 250 Hz and was based on the monitoring of the interrogator scan frequency for the system.

## Bridge description and strain gage installation

**Figure 1.**
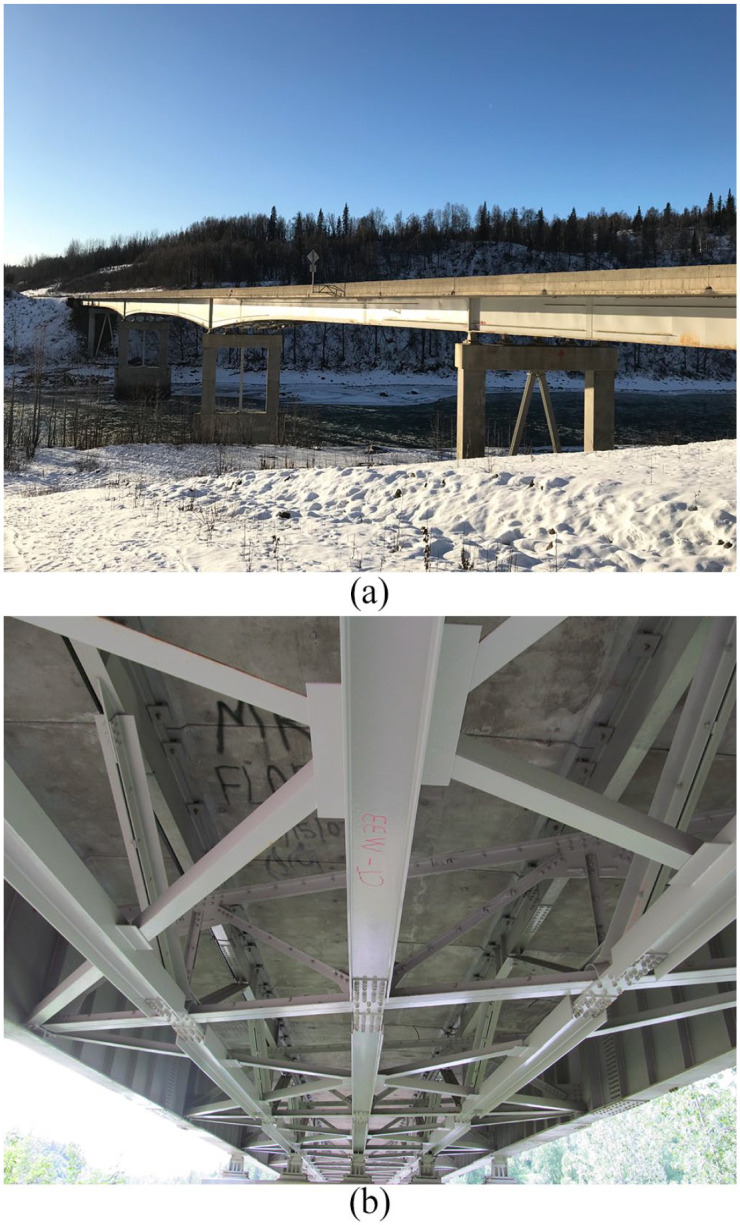
Photograph of the (a) Chulitna river bridge and (b) lower chords.

The 790-foot (241 m) Chulitna River Bridge (Figure 1(a)) is a five-span, steel-girder composite bridge, and this bridge is located on the Parks Highway between Fairbanks and Anchorage in Alaska. The bridge deck is 42 ft 2 in (12.9 m) wide, and the bridge is constructed with two outside steel plate girders and three composite truss structures. The composite truss uses stringers as the top chords and the lower chords, as shown in Figure 1(b).

**Figure 2.**
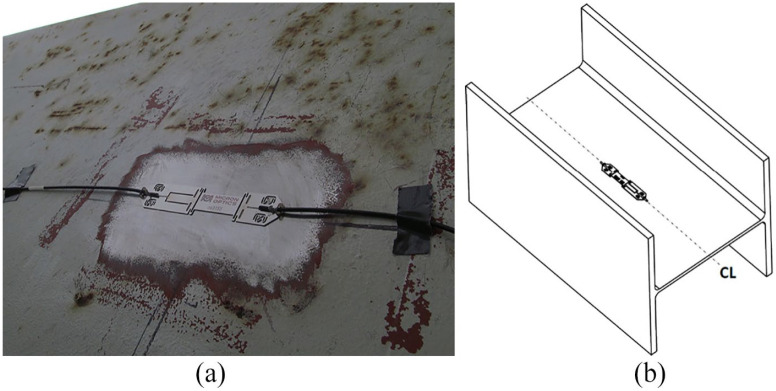
FBG strain gage: (a) strain gage installation and (b) strain gage on the lower chord member.

**Figure 3.**
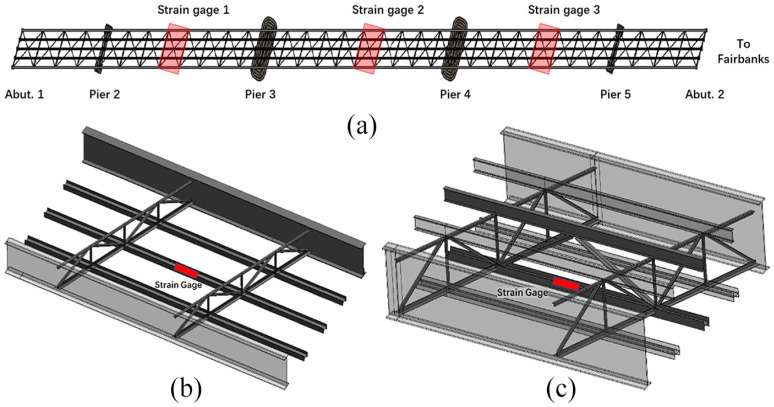
Strain gage installation location: (a) bridge plan view, (b) strain gages 1 and 3 locations, and (c) strain gage 2 location.

The FBG strain gages are welded on the steel structures (Figure 2(a)). In this study, three strain gages are installed on the lower chord members (Figure 2(b)). Strain gages 1, 2, and 3 are located in the middle of different spans (Figure 3(a)). Figure 3(b) shows the location of strain gage 2. Figure 3(c) shows the locations of gages 1 and 2.

## Load test and dynamic parameter identification

Two dump trucks were used to excite the bridge. During the load test, the bridge was closed to other traffic and the trucks crossed the bridge side-by-side in the Fairbanks direction at a speed of 45 mph (72.4 km/h). The bridge was closed until the vibrations were damped out.

**Figure 4.**
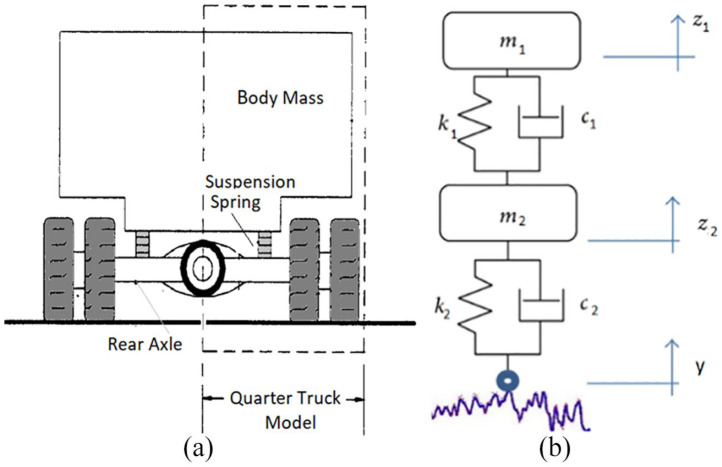
A quarter truck model.

To estimate the excitation or dynamic loading of the truck on the bridge,^30–35^ a quarter truck model, as shown in Figure 4, was deployed, which had following parameters: *m*_1_ = 4500 kg, *m*_2_ = 650 kg, *k*_1_ = 570 kN/m, *k*_2_ = 3000 kN/m, *c*_1_ = 21.07 kN s/m, and *c*_2_ =4 kN s/m. The equation of motion for the system is given as follows

(1) $$\begin{array}{l} \left[\begin{array}{cc} m_{1} & 0\\ 0 & m_{2} \end{array}\right]\left\{\begin{array}{c} {\overset{\cdot \cdot }{Z}}_{1}\left(t\right)\\ {\overset{\cdot \cdot }{Z}}_{1}\left(t\right) \end{array}\right\}+\left[\begin{array}{cc} c_{1} & -c_{1}\\ -c_{1} & c_{1}+c_{2} \end{array}\right]\left\{\begin{array}{c} \dot{Z_{1}}\left(t\right)\\ \dot{Z_{2}}\left(t\right) \end{array}\right\}+\left[\begin{array}{cc} k_{1} & -k_{1}\\ -k_{1} & k_{1}+k_{2} \end{array}\right]\left\{\begin{array}{c} Z_{1}\left(t\right)\\ Z_{2}\left(t\right) \end{array}\right\}\\ =\left\{\begin{array}{c} 0\\ c_{2}\dot{y}\left(t\right)+k_{2}y\left(t\right) \end{array}\right\} \end{array}$$

Traditionally, road profiles have been modeled using Gaussian processes or Laplace processes. To quantify road profile excitation *y* in the above equation, a Gaussian model was used to create an excitation road profile using the ISO 8608 standard for a road roughness spectrum. The profile contains a moving average of white noise, which is a convolution of a kernel function with an infinitesimally “white noise” process having a variance equal to the spatial discretization step. Based on the ISO 8608 standard, the road roughness excitation to truck can be classified as varied classes in terms of the specific power spectrum density (PSD). In the ISO 8608 classification, the relationships between the PSD and spatial frequency for different classes of road roughness are approximated using single straight lines with single slopes on a log–log scale. The ISO 8608 standard specifies a fixed waviness for the PSD road profile. The artificial road profiles are generated numerically to represent road roughness^30,31^ and assume that the tested bridge pavement as ISO B-C class, as the inspected road condition is in good condition

(2) $$y\left(x\right)=\sum_{i=0}^{N}\sqrt{2\Delta sS_{y}\left(i\Delta s\right)\sin\left(2\pi i\Delta sx+\phi_{i}\right)}$$

**Figure 5.**
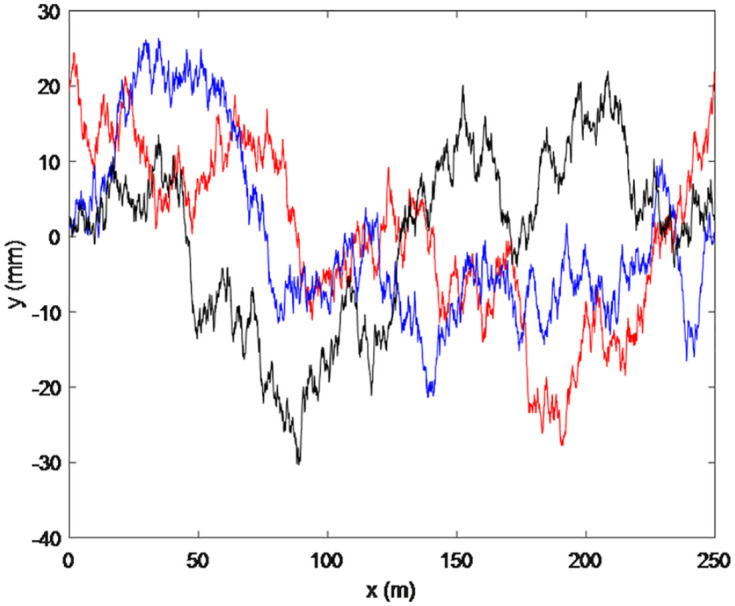
Road profiles generated for the B-C (good pavement) ISO class.

In equation (2), x is a special variable, $S_{y}$ is the PSD from ISO 8608, s is a special frequency, ∆*s* is the frequency band, and $\phi_{i}$ is a random phase angle following a uniform probabilistic distribution within the 0–2π range. Figure 5 shows three typical artificial road profiles generated in three different computations, respectively.

**Figure 6.**
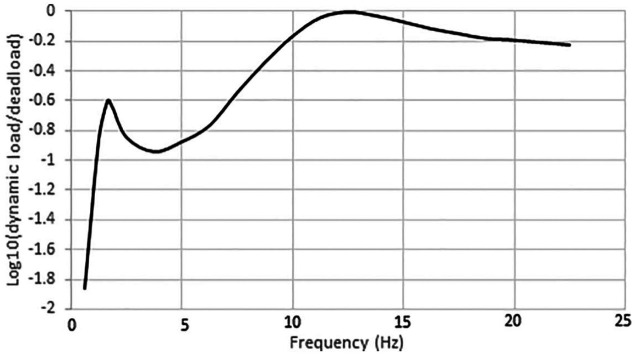
Vehicle load spectrum.

Based on the raw data generated for the road profile *y*(*x*) and the specified speed, the excitation *y*(*t*) is obtained, and equation (1) is solved numerically. The time history of the axle loading on the bridge, $F(t)=c_{2}[{\dot{Z}}_{2}(t)-\dot{y}(t)]+k_{2}[Z_{2}(t)-y(t)]$ , can be obtained, and the spectrum can be calculated. Figure 6 shows the average load spectrum for the truck.

**Figure 7.**
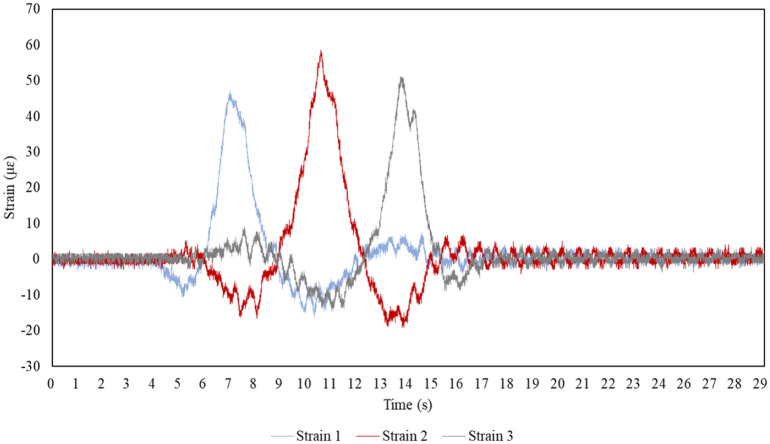
Strain gage reading.

Figure 7 shows the readings from the three FBG strain gages in response to the loading from the truck and the location of the sensors shown in Figure 3. In Figure 7, the strain gage reading is separated into three phases during which the truck approaches the bridge, the truck drives on the bridge, and the truck leaves the bridge.

**Figure 8.**
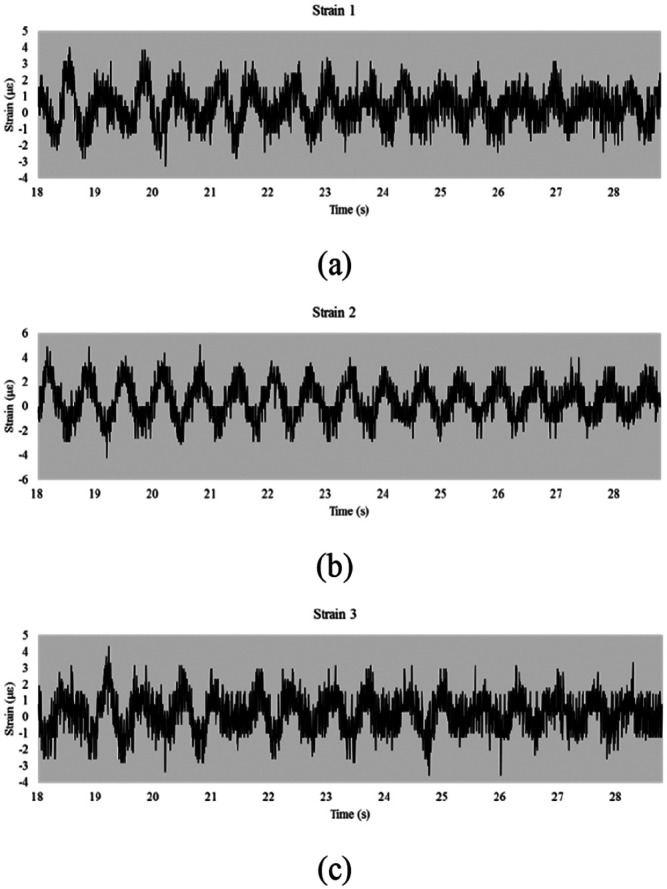
Strain gage reading for trucks leaving the bridge: (a) strain gage 1 signal, (b) strain gage 2 signal, and (c) strain gage 3 signal.

**Figure 9.**
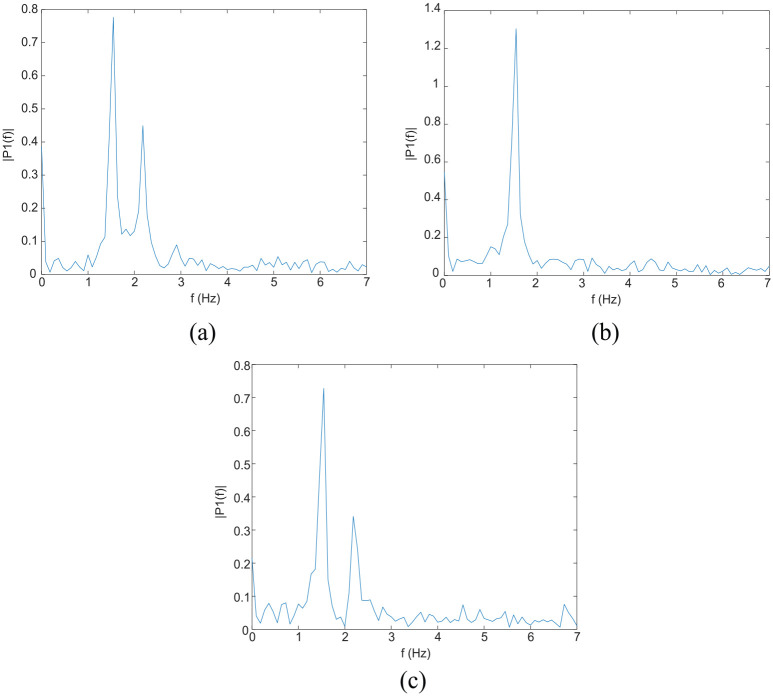
Fast Fourier transform: (a) strain gage 1 reading, (b) strain gage 2 reading, and (c) strain gage 3 reading.

**Figure 10.**
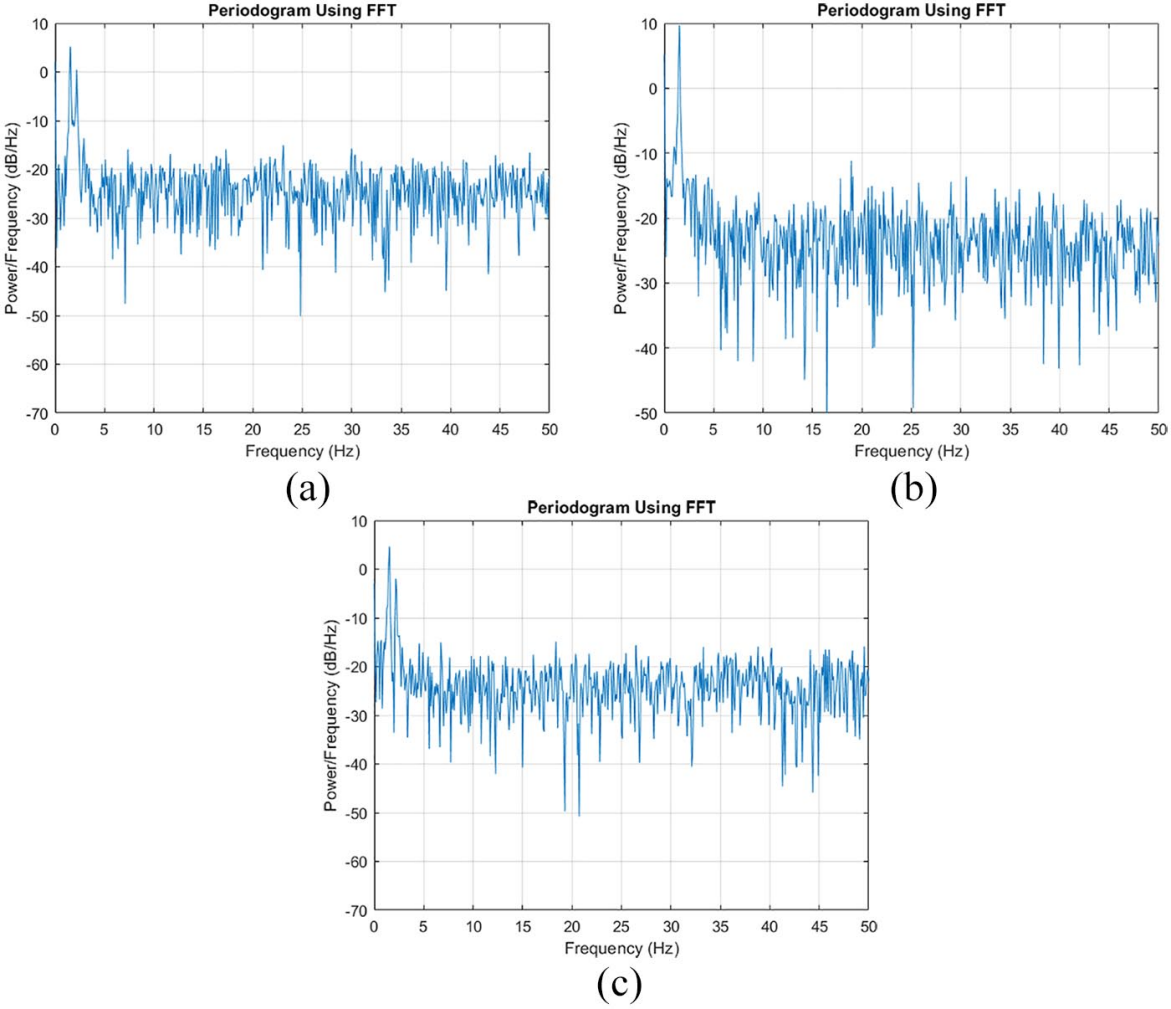
Periodogram power spectrum density: (a) strain gage 1 reading, (b) strain gage 2 reading, and (c) strain gage 3 reading.

Figure 8 depicts the third phase reading, the free decay vibration phase, from Figure 7. Data from strain gages 1, 2, and 3 are shown in Figure 8(a)–(c). In this study, the phase from the free decay vibration was used for dynamic parameter identification by converting it from the time domain to the frequency domain. Figure 9 is the fast Fourier transform (FFT) of the strain gage reading, corresponding to Figure 8. Strain gages 1 (Figure 9(a)) and 3 (Figure 9(c)) identified natural frequencies at 1.546 and 2.180 Hz. Strain gage 2 (Figure 9(b)) only identified the natural frequency at 1.546 Hz. In addition, the corresponding power spectrum shown in Figure 10 is consistent with the results from FFT.

**Figure 11.**
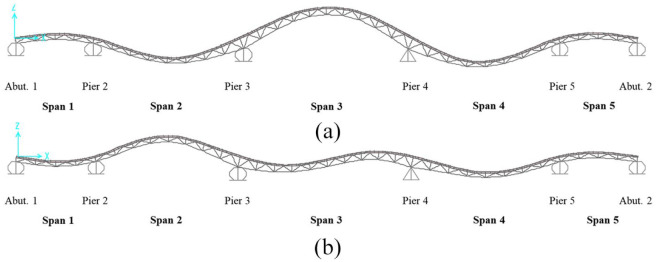
Mode shapes from the FEM analysis: (a) the frequency is 1.584 Hz and (b) the frequency is 2.262 Hz.

To verify that the dynamic parameters were identified using the strain gages, the natural frequencies were compared with the results from a finite element model (FEM) of the bridge. The model was developed using SAP2000 based on the as-built condition. The bridge deck, stringers, and girders were modeled using shell elements, and the truss structures were simulated using frame elements. The bridge had a fixed bearing condition at pier 4. Abutment 1, abutment 2, pier 2, pier 3, and pier 5 are expansion bearing supports. Figure 11 shows the composite truss structure’s mode shapes identified using numerical mode analyses. The frequency in Figure 11(a) is 1.584 Hz (mode shape 1) and that in Figure 11(b) is 2.262 Hz (mode shape 2).

The analytical results were compared with the natural frequencies identified using the strain gages. The experimental results were 1.546 and 2.180 Hz, and the differences between the analytical and experimental results were 2.40% and 3.63% and suggest that the FBG strain gages can provide accurate natural frequencies for dynamic identification.

**Figure 12.**
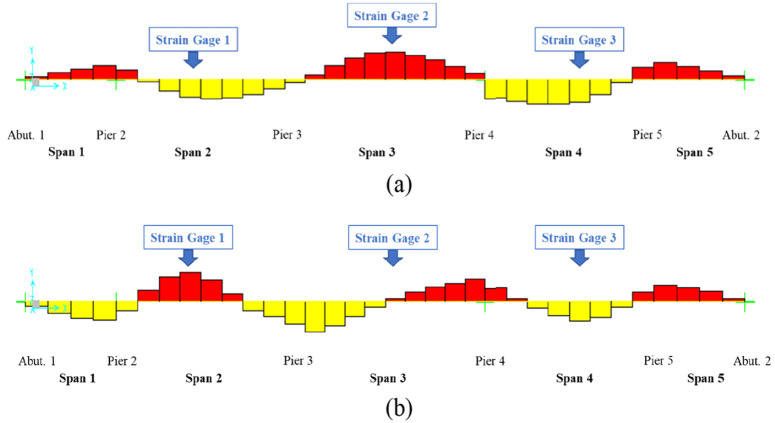
FEM analysis results depicting axial forces on the lower chords: (a) mode shape 1 and (b) mode shape 2.

Figure 12 depicts the axial forces on the lower chords for mode shapes 1 and 2. The strain gages were installed at the middle of spans 2, 3, and 4. In Figure 12(a), large axial loads are seen at the middle of spans 2, 3, and 4. Strain gages 1, 2, and 3 at those locations successfully identified the mode 1 frequency of 1.546 Hz (Figure 9(a)–(c)).

In Figure 12(b), there are also large axial loadings at the middle of spans 2 and 4, and the corresponding frequency of 2.262 Hz was identified using strain gages 1 (Figure 9(a)) and 3 (Figure 9(c)). However, the middle of span 3 has a very small axial load using mode 2; therefore, it was difficult to correlate the numerically and experimentally determined natural frequencies based on the strain measurements at this location. Thus, only one peak was identified (Figure 9(b)).

Moreover, from the vehicle load spectrum shown in Figure 6, we can see that the spectrum has a salient peak at approximately 1.65 Hz and a flat region at 12.5 Hz. Through comparison of Figure 6 with Figure 9, we can see that the first mode of the vehicle is close to the first mode of the bridge. Thus, severe vibrations in the bridge are excited and enhance the resolution and accuracy of the identification based on the decay response. In contrast, the effect of the second mode of the truck on the free decay response of the bridge is not visible in the response spectrum and suggests that the effect is trivial or decays rapidly.

## Conclusion

This study introduced an innovative approach to identifying the dynamic features of a bridge based on strain gage readings. FBG strain gages were installed on the lower chord members of a long-span, steel-girder, composite bridge, and the natural frequencies and mode shapes were successfully identified. The differences between analytical and measured frequencies were below 3.63%. In addition, FEM analyses of the mode shapes due to axial forces correlated with the frequencies identified using dynamic strain measurements. The correlation with numerical analysis supported the utility of using strain data for dynamic frequency identification of bridges. The strain gage readings enable us to estimate global structural vibrations as well as local strains. The present method can be broadly applied for vibration sensing in bridges.
